# Associations Between Chocolate Consumption and Cardiometabolic Risk Markers Across Metabolic Profiles: Insights from a Population-Based Study

**DOI:** 10.3390/nu18183023

**Published:** 2026-09-16

**Authors:** Beatriz Martín-Carro, Estefanía Iglesias-Colino, Darian Montes-Riesgo, Sara Cascón, Leticia Nieto-García, Alfonso Romero, Pablo Pérez-Sánchez, Antonio Sánchez-Puente, Irene Varas-Marcos, Baltasara Blázquez, Candelas Pérez del Villar, David Cembrero-Fuciños, Paz Muriel, José Carlos Moyano-Maza, Inmaculada Santolino, Amalia Martín-Gallego, Lydia González-González, Javier Maíllo-Seco, María José Ruiz-Olgado, Luis M. Rincón, María Isidoro-García, Pedro L. Sánchez

**Affiliations:** 1 Department of Cardiology, University Hospital of Salamanca, 37007 Salamanca, Spainplsanchez@saludcastillayleon.es (P.L.S.); 2 Instituto de Investigación Biomédica de Salamanca (IBSAL), 37007 Salamanca, Spain; 3 Centro de Investigación Biomédica en Red de Enfermedades Cardiovasculares (CIBER-CV), Instituto de Salud Carlos III, 28029 Madrid, Spain; 4 Faculty of Medicine, University of Salamanca, 37007 Salamanca, Spain; 5 Department of Internal Medicine, Complejo Asistencial de Zamora, 49022 Zamora, Spain; 6 Robleda Primary Care Center, 37521 Salamanca, Spain; 7 Miguel Armijo Primary Care Centre, 37007 Salamanca, Spain; 8 Miranda del Castañar Primary Care Centre, 37660 Salamanca, Spain; 9 Clinical Laboratory Department, University Hospital of Salamanca, 37007 Salamanca, Spain; 10 La Alamedilla Primary Care Centre, 37003 Salamanca, Spain; 11 Santa Marta Primary Care Centre, 37900 Salamanca, Spain; 12 Department of Cardiology, Complejo Asistencial de Zamora, 49022 Zamora, Spain

**Keywords:** chocolate, cardiometabolic risk markers, diabetes, hypertension, dyslipidemia, population-based study

## Abstract

**Background/Objectives**: Studies evaluating the association between chocolate consumption and cardiometabolic health have reported inconsistent findings, and whether these associations differ according to underlying metabolic status remains unclear. This cross-sectional study analysed whether metabolic profile modifies the associations between chocolate consumption and cardiometabolic risk markers in 1960 adults from the SALMANTICOR population-based cohort. **Methods**: Chocolate consumption was classified by chocolate type (dark or milk) and frequency of intake (occasional: 1–3 days/week; regular: ≥4 days/week). Multivariable linear regression models were used to evaluate associations with fasting glucose, glycated haemoglobin (HbA1c), high-density lipoprotein (HDL) cholesterol, low-density lipoprotein (LDL) cholesterol, triglycerides, body mass index (BMI), waist circumference, and systolic blood pressure (SBP), with additional interaction analyses for diabetes, hypertension, and dyslipidaemia. Models were adjusted for age, sex, BMI (except when BMI was the outcome), smoking, alcohol consumption, physical activity, olive oil intake, and commercial pastry consumption. **Results**: Overall, 30.1% of participants reported habitual chocolate consumption. In multivariable models without interaction terms, both dark and milk chocolate consumption were associated with lower BMI, whereas dark chocolate consumption was associated with higher LDL cholesterol. After Bonferroni correction for multiple comparisons, diabetes modified the associations of dark chocolate consumption with fasting glucose and HbA1c and of milk chocolate consumption with HbA1c. Among individuals with diabetes, dark chocolate consumption was associated with lower fasting glucose and HbA1c, whereas milk chocolate consumption was associated with lower HbA1c compared with non-consumption. No such associations were observed among individuals without diabetes. **Conclusions**: These findings suggest that the association between chocolate consumption and cardiometabolic health is not uniform across the population but differs according to the underlying metabolic profile. In particular, the associations with glycaemic markers were confined to individuals with diabetes, suggesting that metabolic status may modify the relationship between habitual chocolate consumption and glycaemic control. Further prospective population-based studies and intervention trials are needed to confirm these findings.

## 1. Introduction

Chocolate is widely consumed worldwide, and dark chocolate is among the richest dietary sources of cocoa-derived polyphenols, including flavan-3-ols (catechin and epicatechin), anthocyanins, and procyanidins [1,2,3]. These bioactive compounds have attracted considerable interest because of their antioxidant, anti-inflammatory, and vasodilatory properties, largely mediated through improvements in nitric oxide bioavailability and endothelial function [4,5,6,7].

Experimental evidence suggests that cocoa flavanols may influence cardiometabolic health through multiple interconnected mechanisms. Improvements in endothelial function and vascular tone may enhance tissue perfusion and facilitate insulin and nutrient delivery to peripheral tissues [8,9]. In addition, cocoa flavanols have been shown to attenuate oxidative stress and chronic low-grade inflammation through the inhibition of nuclear factor kappa B (NF-κB) signalling and the reduction in circulating inflammatory mediators, including tumour necrosis factor alpha (TNF-α), interleukin-6 (IL-6), and C-reactive protein [7,10,11,12]. Collectively, these mechanisms may contribute to improved glucose homeostasis, lipid metabolism, and blood pressure regulation [13,14,15].

Consistent with these biological mechanisms, randomized controlled trials and meta-analyses have reported beneficial effects of cocoa or chocolate consumption on total cholesterol and low-density lipoprotein (LDL) cholesterol [13,14], fasting glucose [10,15], and systolic and diastolic blood pressure [16,17,18]. However, the results have not been consistent across studies, with some reporting null or less favourable associations for specific outcomes. Differences in the populations studied, chocolate formulations, cocoa flavanol dose, intervention duration, and study design may contribute to this heterogeneity.

Interpreting the available evidence is further complicated by the considerable diversity of chocolate products consumed under real-world conditions. Compared with milk chocolate, dark chocolate generally contains higher concentrations of cocoa flavanols and lower amounts of added sugars, whereas milk proteins and the higher sugar content of milk chocolate may reduce flavanol bioavailability and biological activity [19]. Although many studies in recent years have examined the association between chocolate consumption and cardiometabolic outcomes, most have assessed dark chocolate intake without comparing or analysing milk chocolate or considering the frequency of consumption. Furthermore, few population-based studies have investigated whether these associations vary according to underlying metabolic status.

Metabolic disorders such as diabetes, hypertension, and dyslipidaemia are characterized by endothelial dysfunction, chronic low-grade inflammation, insulin resistance, and altered lipid metabolism [20,21]. These pathophysiological alterations may modify the response to cocoa-derived bioactive compounds, suggesting that the associations between chocolate consumption and cardiometabolic risk markers could differ according to metabolic status [22]. However, this effect modification has received little attention in population-based studies. We hypothesized that the associations between chocolate consumption and cardiometabolic risk markers would vary according to chocolate type and consumption frequency and would be modified by underlying metabolic status.

Therefore, the aim of the present study was to analyse whether the associations between chocolate consumption, according to chocolate type and consumption frequency, and cardiometabolic risk markers differ according to metabolic status in a population-based cohort. Specifically, we assessed effect modification by diabetes, hypertension, and dyslipidaemia.

## 2. Materials and Methods

### 2.1. Study Design and Participants

This cross-sectional analysis used baseline data from the SALMANTICOR Study (ClinicalTrials.gov Identifier: NCT03429452), a population-based cohort conducted in Salamanca, Spain. Between 2015 and 2018, a total of 2063 adults aged ≥ 18 years were recruited using stratified random sampling by sex, age, and geographic area. The sampling strategy was designed to obtain a representative sample of the adult population of Salamanca. Detailed study design and procedures have been previously published [23].

For the present analysis, participants with missing data on chocolate consumption were excluded, resulting in a final sample of 1960 individuals (95% of the cohort).

### 2.2. Dietary Assessment and Chocolate Consumption

Dietary habits were assessed using a structured questionnaire administered by trained interviewers during face-to-face visits conducted at the time of the physical examination. Participants reported the type of chocolate usually consumed (dark, milk, or white chocolate) and their weekly consumption frequency. Only 8 participants reported consuming white chocolate; therefore, these participants were included in the milk chocolate category for the present analyses because the small number of white chocolate consumers precluded a separate statistical analysis of this category. Three participants reported consuming both dark and milk chocolate and were therefore included in both chocolate-type categories. Thus, the dark and milk chocolate groups were not mutually exclusive, and each chocolate type was analysed independently against non-consumption. The questionnaire also collected detailed information on lifestyle and dietary behaviours, including smoking, alcohol consumption, physical activity, meal patterns, olive oil intake, pastry consumption, tea and meat intake, nutritional label-reading habits, and medication use. All data were collected at baseline during the same visit following standardized procedures. Self-reported diagnoses were verified by primary care physicians according to internationally accepted diagnostic criteria.

### 2.3. Clinical Measurements

Anthropometric measurements, including body weight, height, and waist circumference, were obtained using standardized procedures. Body mass index (BMI) was calculated as weight (kg) divided by height squared (m^2^). Systolic and diastolic blood pressure (SBP and DBP) were measured with validated automatic devices.

Arterial stiffness was assessed by the Cardio-Ankle Vascular Index (CAVI) using the VaSera VS-1500 device (Fukuda Denshi, Tokyo, Japan).

Information on the presence or absence of diabetes, hypertension, dyslipidaemia, other comorbidities, and current pharmacological treatments was obtained through structured interviews, review of electronic medical records, and clinical assessment. Information on diabetes type was not available in the database.

### 2.4. Blood Biochemical Analysis

Fasting blood samples were collected after at least 12 h of fasting and abstinence from nicotine, alcohol, and caffeine. Serum and plasma were separated by centrifugation at 3500 rpm for 5 min and stored at −80 °C until analysis.

Plasma concentrations of *N*-terminal pro-B-type natriuretic peptide (NT-proBNP), growth differentiation factor 15 (GDF15), ferritin, and high-sensitivity troponin T (hs-TnT) were determined by electrochemiluminescence immunoassay (ECLIA), using ELECSYS^®^ reagents (Roche Diagnostics; Mannheim, Germany) on a cobas^®^ e601 analytical module. The limits of detection for these assays were 5 pg/mL for NT-proBNP, 400 ng/mL for GDF15, 0.5 ng/mL for ferritin and 1.3 ng/L for hs-TnT.

Plasma concentrations of glucose, high-sensitivity C-reactive protein (hs-CRP), total cholesterol, HDL cholesterol, LDL cholesterol, triglycerides, creatinine, iron, and transferrin were determined using photometric, immunoturbidimetric and indirect potentiometry with ion-selective electrodes (ISE) methods on cobas^®^ c501 analyzers (Roche Diagnostics). Glycated haemoglobin (HbA1c) was measured by high-performance liquid chromatography (HPLC) on HA-8160 analyzers (Menarini Diagnostics; Florence, Italy).

All analytical measurements were performed at the Clinical Laboratory Department of the University Hospital of Salamanca, an ISO 15189:2022-accredited laboratory [24]. Calibration and quality control procedures followed the manufacturers’ recommendations, and laboratory personnel were blinded to participants’ clinical information.

### 2.5. Statistical Analysis

Continuous variables were presented as median and interquartile range (IQR) due to the non-normal distribution and categorical variables as percentages. Differences in participant characteristics across chocolate consumption categories were assessed using the non-parametric Kruskal–Wallis test for continuous variables and Chi-squared test for categorical variables.

Multivariate linear regression models were used to analyse associations between chocolate consumption and cardiometabolic risk markers, including fasting glucose, HbA1c, HDL cholesterol, LDL cholesterol, triglycerides, BMI, waist circumference, and SBP.

Chocolate consumption was evaluated according to: (1) Chocolate type, classified as dark or milk chocolate consumption (yes vs. no). The dark and milk chocolate groups were not mutually exclusive, as the three participants reporting consumption of both types were included in both categories. Associations for each type were estimated separately using non-consumption as the reference category. (2) Consumption frequency, categorised as non-consumers, occasional consumers (1–3 days/week), and regular consumers (≥4 days/week). Analyses of consumption frequency were restricted to chocolate consumers with available frequency data, using occasional consumers as the reference group.

Continuous outcome variables were standardised to z-scores before analysis to facilitate comparison of regression coefficients across outcomes with different units of measurement.

Models were adjusted for age, sex, BMI (except when BMI was the outcome variable), smoking status, alcohol intake, physical activity, olive oil consumption, and commercial pastry consumption. Covariates were selected a priori based on their established associations with cardiometabolic outcomes and their potential role as confounders.

Effect modification by diabetes, hypertension, and dyslipidaemia was assessed by including multiplicative interaction terms between each chocolate exposure (type or consumption frequency) and each metabolic condition in the multivariable regression models. A total of 48 interaction tests were evaluated for each analysis (8 outcomes × 2 chocolate types × 3 metabolic conditions). Bonferroni correction was applied to account for multiple testing, with the adjusted significance threshold defined as pBonferroni < 0.05, corresponding to an unadjusted threshold of α = 0.05/48 = 0.0010417. When interaction terms remained statistically significant after Bonferroni correction, simple effects were estimated and graphically represented.

For the primary regression analyses and simple effects, two-tailed *p*-values < 0.05 were considered statistically significant, and 95% confidence intervals (CIs) were reported. For interaction terms, statistical significance was determined using the Bonferroni-adjusted threshold (pBonferroni < 0.05). Analyses were performed using R software version 4.4.3 (R Foundation for Statistical Computing, Vienna, Austria).

### 2.6. Ethical Considerations

The study was conducted in accordance with the Declaration of Helsinki and approved by the Clinical Research Ethics Committee of the Salamanca Health Area (approval number PI 2014 09 01; 29 September 2014, Salamanca, Spain). All participants provided written informed consent.

## 3. Results

### 3.1. Baseline Characteristics

The main baseline characteristics of the study population according to chocolate consumption frequency are detailed in Table 1. Overall, 30.1% of participants reported chocolate consumption. Compared with non-consumers, chocolate consumers were younger (median age, 52 [interquartile range (IQR): 41-62] vs. 59 (46–71) years; *p* < 0.001). Chocolate consumption was also more common among women than men, with 35.6% of women and 23.6% of men reporting chocolate consumption (*p* < 0.001).

Non-consumers had higher body weight, body mass index (BMI), waist circumference, systolic and diastolic blood pressure (SBP and DBP), and arterial stiffness (all *p* < 0.01).

Regarding lifestyle characteristics, chocolate consumers reported lower alcohol intake, higher tea consumption and greater adherence to certain health-related dietary behaviours, including more frequent nutritional label reading and less frequent consumption of commercial pastries (*p* < 0.001). No significant differences were observed in smoking status or physical activity levels.

Non-consumers had a higher prevalence of diabetes and hypertension and were more frequently treated with antihypertensive and hypoglycaemic drugs (*p* < 0.001). Overall medication use was also more frequent among non-consumers (*p* < 0.05). In addition, chronic kidney disease, previous stroke or transient ischemic attack (TIA), heart failure, and valvulopathy were significantly less frequent among regular chocolate consumers (*p* < 0.05). No significant differences were observed for the remaining comorbidities.

Several biochemical markers differed across chocolate consumption categories (Table 2), including fasting glucose, HbA1c, total cholesterol, high-density lipoprotein (HDL) cholesterol, triglycerides, high-sensitivity cardiac troponin T (hs-TnT), high-sensitivity C-reactive protein (hs-CRP), growth differentiation factor 15 (GDF15) and creatinine, with non-consumers generally showing a less favourable cardiometabolic profile. By contrast, LDL cholesterol (LDL) and NT-proBNP concentrations did not differ significantly between groups.

### 3.2. Associations Between Dark or Milk Chocolate Consumption and Cardiometabolic Risk Markers

**Figure 1:**
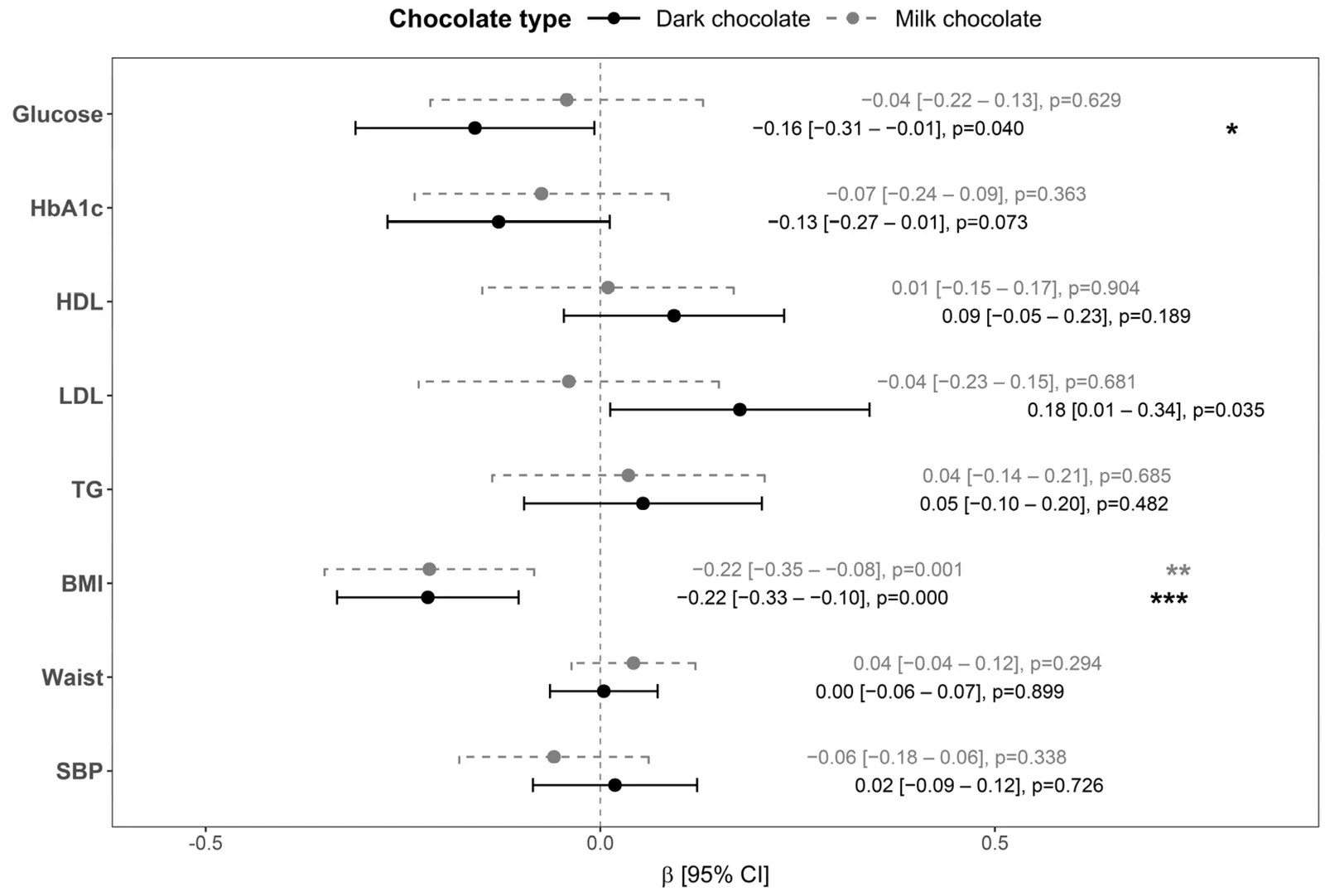
Associations of dark and milk chocolate consumption with cardiometabolic risk markers in the overall cohort. Points represent multivariable-adjusted standardized β coefficients and horizontal bars indicate 95% confidence intervals (CI), using non-consumption as the reference category. Black and grey symbols represent dark and milk chocolate consumption, respectively. Models were adjusted for age, sex, body mass index (BMI; except when BMI was the outcome), smoking status, alcohol consumption, physical activity, olive oil intake, and pastry consumption. * *p* < 0.05; ** *p* < 0.01, *** *p* < 0.001. HbA1c, glycated haemoglobin; HDL, high-density lipoprotein cholesterol; LDL, low-density lipoprotein cholesterol; TG, triglycerides; SBP, systolic blood pressure.

Associations between chocolate consumption according to type (dark or milk chocolate) and cardiometabolic risk markers in the overall cohort are shown in Figure 1. After multivariable adjustment, dark chocolate consumption was associated with lower fasting glucose and higher LDL-cholesterol compared with non-consumption (both *p* < 0.05). Consumption of both dark and milk chocolate was associated with lower BMI (both *p* < 0.001). No significant associations were observed for HbA1c, HDL cholesterol, triglycerides, waist circumference, or systolic blood pressure.

### 3.3. Chocolate Type and Cardiometabolic Risk Markers: Effect Modification by Metabolic Status

Effect modification by diabetes, hypertension, and dyslipidaemia was evaluated by including interaction terms in the multivariable regression models. Interaction *p*-values are presented in Table 3, and the fully adjusted regression models are shown in Supplementary Table S1.

Diabetes significantly modified the associations of dark chocolate consumption with fasting glucose and HbA1c (both unadjusted *p*-values < 0.001; pBonferroni < 0.001 and =0.001, respectively). Similarly, diabetes also modified the association of milk chocolate consumption with HbA1c (unadjusted *p*-value < 0.001; pBonferroni = 0.009). No other interaction terms were statistically significant after Bonferroni correction.

**Figure 2:**
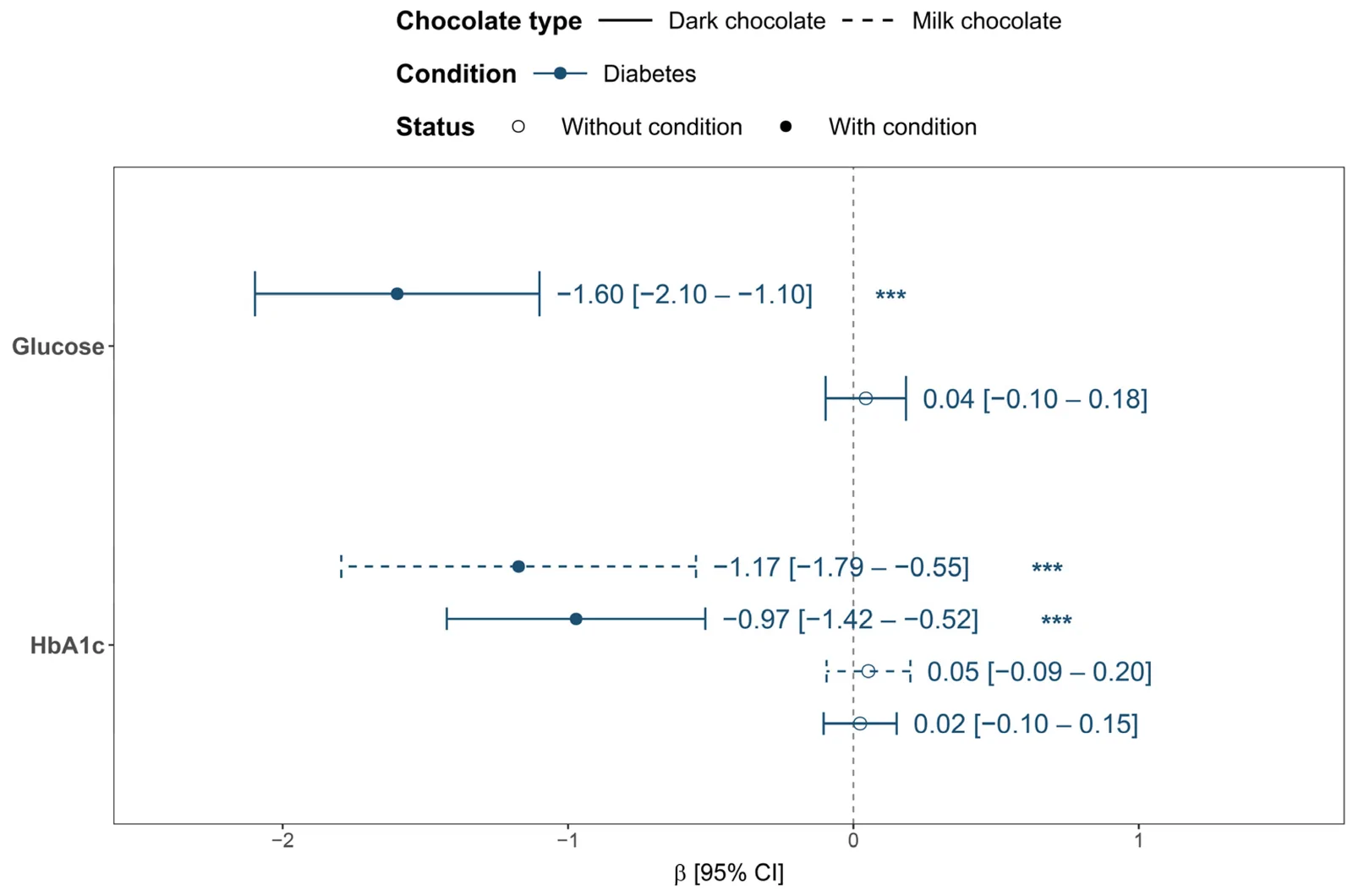
Estimated simple effects of dark and milk chocolate consumption on cardiometabolic risk markers according to diabetes status for interactions significant after Bonferroni correction. Points represent multivariable-adjusted standardized β coefficients and horizontal bars indicate 95% confidence intervals (CI). Filled and open symbols represent participants with and without diabetes. Solid and dashed lines represent dark and milk chocolate consumption, respectively. Reference category: no consumption. Models were adjusted for age, sex, body mass index (BMI; except when BMI was the outcome), smoking status, daily alcohol consumption, physical activity level, olive oil intake, and commercial pastry consumption. *** *p* < 0.001. BMI, body mass index; HbA1c, glycated haemoglobin.

Estimated simple effects for the interactions that remained statistically significant after Bonferroni correction are presented in Figure 2. Among individuals with diabetes, dark chocolate consumption was significantly associated with lower fasting glucose and HbA1c compared with non-consumption (*p* < 0.001 and <0.01, respectively). Similarly, among individuals with diabetes, milk chocolate consumption was associated with lower HbA1c compared with non-consumption (*p* < 0.05). In contrast, among individuals without diabetes, neither dark chocolate consumption nor milk chocolate consumption was significantly associated with fasting glucose or HbA1c.

### 3.4. Chocolate Consumption Frequency and Cardiometabolic Risk Markers: Effect Modification by Metabolic Status

Effect modification of the associations between chocolate consumption frequency (regular vs. occasional) and cardiometabolic risk markers by diabetes, hypertension, and dyslipidaemia was assessed through interaction terms in multivariable linear regression models. Interaction *p*-values are presented in Table 4, and the fully adjusted regression model results are provided in Supplementary Table S2.

Before Bonferroni correction, interactions between diabetes and dark chocolate consumption frequency were observed for fasting glucose (*p* = 0.028), LDL cholesterol (*p* = 0.008), and triglycerides (*p* = 0.034). However, none of these interactions remained statistically significant after Bonferroni correction. No significant interactions terms were observed for hypertension or dyslipidaemia, or for milk chocolate consumption frequency with any of the metabolic conditions.

## 4. Discussion

The present study showed that the associations between habitual chocolate consumption and glycaemic markers differed according to diabetes status. Among individuals with diabetes, dark chocolate consumption was associated with lower fasting glucose and HbA1c, while milk chocolate consumption was associated with lower HbA1c compared with non-consumption. These findings suggest that the association between habitual chocolate consumption and glycaemic control may depend on baseline metabolic characteristics rather than being uniform across the population.

The inverse associations between chocolate consumption and glycaemic markers confined to individuals with diabetes support the hypothesis that the relationship between chocolate intake and glucose homeostasis may depend on the underlying metabolic context. Cocoa-derived bioactive compounds have been proposed to improve glucose metabolism through several mechanisms, including enhancement of endothelial function and insulin sensitivity, increased nitric oxide bioavailability, and modulation of oxidative stress and inflammatory pathways involved in metabolic dysfunction [8,25,26,27,28]. These mechanisms may be especially relevant in individuals with diabetes, in whom endothelial dysfunction, insulin resistance, and chronic low-grade inflammation are more pronounced [29]. Accordingly, individuals with greater metabolic impairment may derive larger physiological benefits from cocoa-derived compounds than metabolically healthier individuals. However, alternative explanations should also be considered.

Most previous evidence on chocolate and cardiometabolic health has been derived from intervention studies using standardised cocoa flavanol preparations or high-cocoa-content chocolate, whereas evidence from observational studies assessing habitual consumption patterns remains comparatively limited. Moreover, the flavanol-rich cocoa products evaluated in intervention studies differ substantially from commercially available chocolate consumed in real-world settings, limiting the direct comparability between intervention and observational evidence. Furthermore, few population-based studies have simultaneously evaluated chocolate type and consumption frequency, making it difficult to determine whether these distinct aspects of chocolate consumption are differentially associated with cardiometabolic markers [30]. Evidence from intervention studies specifically conducted in individuals with type 2 diabetes has also been heterogeneous, with some studies reporting favourable effects on glycaemic or cardiometabolic markers and others showing little or no effect [31,32,33]. Consequently, it remains uncertain whether the benefits reported in intervention trials translate into habitual dietary behaviours. The fact that associations were observed for both dark and milk chocolate further argues against attributing the findings exclusively to cocoa flavanol exposure. Differences in overall dietary patterns, consumer characteristics, habitual portion size, and the composition of commercially available chocolate products are also likely to have contributed.

The possibility that the observed associations reflect differences in the clinical and behavioural context of individuals with diabetes should also be considered. Individuals with diabetes may have changed their chocolate consumption after diagnosis as part of disease management. For example, individuals with diabetes who consume chocolate may also have better overall dietary quality, greater health awareness, or more frequent healthcare contact, all of which could confound the observed associations and may not be fully captured by the covariates included in our models. In our cohort, chocolate consumption was less prevalent among participants with diabetes than among those without diabetes (13.6% vs. 32.3%, *p* < 0.001). This difference was observed for both dark chocolate (9.3% vs. 18.2%, *p* < 0.001) and milk chocolate (4.2% vs. 14.3%, *p* < 0.001). However, among consumers, the reported frequency of consumption did not differ according to diabetes status, either for dark chocolate (3.5 [2.0–5.8] vs. 3.0 [2.0–5.0] days/week, *p* = 0.875) or milk chocolate (3.0 [2.0–4.0] vs. 3.0 [2.0–4.2] days/week, *p* = 0.633). This pattern may indicate that chocolate consumption among individuals with diabetes represents a selected dietary behaviour, whereby those who continue to consume chocolate after diagnosis may differ systematically from those who reduce or avoid it. Although several lifestyle and dietary factors were included in the multivariable models, these characteristics may not have been fully captured, leaving the possibility of residual confounding. Diabetes treatment may represent an additional source of heterogeneity, as glucose-lowering therapies influence glycaemic markers and may be accompanied by dietary counselling and behavioural changes. In addition, the cross-sectional design prevents determining whether chocolate consumption preceded differences in glycaemic markers or whether individuals with better glycaemic control were more likely to consume chocolate.

In the analyses performed in the overall cohort without accounting for effect modification by metabolic status, consumption of both dark and milk chocolate was associated with lower BMI. This finding is consistent with previous observational studies reporting lower adiposity among chocolate consumers [34,35,36]. Several mechanisms have been proposed, including favourable effects of cocoa flavanols on satiety regulation, energy expenditure, oxidative stress, and adipose tissue metabolism [37,38,39,40]. Nevertheless, this association should be interpreted cautiously, as it may be particularly susceptible to residual confounding. In our cohort, chocolate consumers were younger and displayed healthier lifestyle characteristics, including lower alcohol consumption and lower intake of commercial pastries. Although these factors were included in the multivariable models, chocolate consumption may partly represent a marker of broader dietary and behavioural patterns rather than an independent determinant of adiposity. The associations observed in the overall cohort were modest in magnitude, and their clinical relevance remains uncertain.

Taken together, these findings suggest that the association between chocolate consumption and cardiometabolic health is context-dependent and may vary according to metabolic status rather than representing a uniform association across the general population. The findings that remained statistically significant after correction involved the modification of associations with glycaemic markers by diabetes. Importantly, these observations do not establish a beneficial effect of chocolate consumption in individuals with diabetes but rather indicate that metabolic status may be an important context in which habitual dietary exposures should be interpreted.

From a clinical perspective, these findings should be interpreted cautiously and do not support assuming that chocolate consumption has uniformly beneficial or detrimental effects. In particular, the associations observed for glycaemic markers among individuals with diabetes highlight the importance of underlying metabolic context when interpreting dietary exposures, but they should not be interpreted as evidence that chocolate consumption improves diabetes control. Given the cross-sectional design and the potential for residual confounding, these findings should not be used to support specific dietary recommendations regarding chocolate consumption. Chocolate consumption should therefore be considered within the broader dietary and cardiometabolic context of each individual rather than as a specific strategy for improving glycaemic control.

This study has several strengths. It included a well-characterised population-based cohort with detailed assessment of cardiometabolic risk factors, and allowed simultaneous evaluation of chocolate type, consumption frequency, and potential effect modification by diabetes, hypertension, and dyslipidaemia. This approach enabled the assessment of whether associations between chocolate consumption and cardiometabolic risk markers differed according to clinically relevant metabolic profiles. Furthermore, the simultaneous consideration of multiple metabolic conditions better reflects the complexity of real-world cardiometabolic phenotypes. The use of interaction analyses with correction for multiple testing provided a conservative assessment of effect modification and reduced the likelihood of false-positive findings arising from multiple comparisons.

Several limitations should also be acknowledged. First, the cross-sectional design precludes establishing the temporal sequence between chocolate consumption and cardiometabolic markers and, therefore, causal relationships. Thus, reverse causation cannot be completely excluded, as participants may have modified their dietary habits in response to their health status or other factors. This issue may be particularly relevant in individuals with diabetes, who may change their dietary habits after diagnosis or according to their degree of glycaemic control. Second, chocolate consumption was self-reported and information on cocoa percentage, flavanol content, amount consumed per occasion (grams or servings), and specific product formulation was unavailable. Therefore, the actual exposure to cocoa-derived flavanols could not be characterized, and the observed associations cannot be attributed specifically to flavanol-related effects. Differences in total energy or sugar intake may also have contributed to the observed associations. Likewise, the broad categories of dark and milk chocolate may encompass considerable heterogeneity in cocoa content, processing, sugar, fat, and other ingredients, further limiting inference regarding specific components responsible for the observed associations. Third, despite adjustment for several relevant lifestyle and dietary variables, residual confounding cannot be excluded, including that related to factors such as total energy intake, which was not available in the database. An overall measure of dietary quality was also not available, and differences in other dietary behaviours observed between chocolate consumers and non-consumers may reflect broader differences in dietary patterns that were not fully captured by the variables included in the models. Therefore, the possibility that healthier overall dietary patterns among chocolate consumers contributed to the observed associations cannot be excluded.

The generalizability of our findings may also be limited, as the study was conducted in a Spanish population whose dietary habits and patterns of chocolate consumption may differ from those of other geographical regions. Differences in dietary patterns, as well as in the composition and formulation of commercially available chocolate products, may limit the extrapolation of our findings to other populations. Replication in independent cohorts from different geographical and cultural settings, using comparable definitions of chocolate consumption and detailed assessment of dietary and product characteristics, would be needed to determine whether these findings are reproducible across populations. Taken together, these limitations and the observational nature of the study indicate that the findings should be considered hypothesis-generating rather than a basis for specific dietary recommendations regarding chocolate consumption, even within narrowly defined metabolic populations.

## 5. Conclusions

The present study suggests that associations between chocolate consumption and cardiometabolic risk markers may vary according to underlying metabolic profile rather than being uniform across the general population. Among individuals with diabetes, dark chocolate consumption was associated with lower fasting glucose and HbA1c, whereas milk chocolate consumption was associated with lower HbA1c. These findings suggest that diabetes status may modify the association between chocolate consumption and glycaemic markers.

Given the cross-sectional design, these findings should be interpreted as hypothesis-generating rather than evidence of causality. They should therefore not be interpreted as supporting specific dietary recommendations regarding chocolate consumption. Further prospective population-based studies and intervention trials are needed to determine whether the associations between habitual chocolate consumption and cardiometabolic health differ across metabolic profiles and to establish whether the observed findings reflect true biological effects of chocolate or differences in dietary and lifestyle behaviours.

## Figures and Tables

**Table 1:** Main baseline characteristics of participants overall and according to frequency of chocolate consumption: no consumption, occasional consumption (1–3 times/week) and regular consumption (≥4 times/week).

	**Overall**	**No Consumption**	**Occasional**	**Regular**	***p*-Value **
N	1960	1370	340	250	
Sex (%)					** <0.001 **
Women	1061 (54.1)	683 (49.9)	221 (65.0)	157 (62.8)	
Men	899 (45.9)	687 (50.1)	119 (35.0)	93 (37.2)	
Age (%)	56.00 [44.00, 69.00]	59.00 [46.00, 71.00]	51.00 [41.00, 61.00]	52.50 [40.00, 63.00]	** <0.001 **
**Anthropometric measurements**					
Weight (kg) [median (IQR)]	71.50 [62.00, 81.00]	73.00 [64.00, 82.00]	67.00 [60.00, 78.00]	68.00 [60.00, 78.00]	** <0.001 **
Height (cm) [median (IQR)]	165.00 [160.00, 171.00]	165.00 [160.00, 171.00]	165.00 [159.00, 170.00]	165.50 [160.00, 172.00]	0.116
Waist circumference (cm) [median (IQR)]	89.00 [82.00, 98.00]	91.00 [83.00, 99.00]	86.00 [79.00, 95.00]	86.00 [78.00, 93.00]	** <0.001 **
BMI (kg/m^2^) [median (IQR)]	25.91 [23.39, 28.91]	26.26 [23.89, 29.38]	25.32 [22.50, 28.01]	24.52 [22.03, 27.55]	** <0.001 **
**Blood pressure and hemodynamic parameters**					
SBP (mmHg) [median (IQR)]	138.00 [126.00, 150.00]	140.00 [128.00, 152.00]	134.00 [123.00, 148.00]	133.50 [124.00, 147.00]	** <0.001 **
DBP (mmHg) [median (IQR)]	87.00 [80.00, 94.00]	87.00 [80.00, 94.00]	85.00 [78.00, 92.00]	85.00 [79.00, 92.00]	** 0.008 **
Arterial Stiffness [median (IQR)]	7.70 [6.70, 9.10]	7.90 [6.80, 9.20]	7.40 [6.60, 8.50]	7.50 [6.70, 8.90]	** <0.001 **
**Lifestyle factors**					
Smoker (%)					0.191
Current- smoker	388 (19.8)	282 (20.6)	63 (18.5)	43 (17.2)	
Ex-smoker	557 (28.4)	378 (27.6)	112 (32.9)	67 (26.8)	
Never- smoker	1015 (51.8)	710 (51.8)	165 (48.5)	140 (56.0)	
Daily alcohol consumption = Yes (%)	504 (25.7)	390 (28.5)	62 (18.2)	52 (20.8)	** <0.001 **
Level of physical activity (%)					0.239
High	158 (8.1)	105 (7.7)	31 (9.1)	22 (8.8)	
Moderate	1559 (79.6)	1081 (78.9)	274 (80.8)	204 (81.6)	
Low or inactive	242 (12.4)	184 (13.4)	34 (10.0)	24 (9.6)	
Meal patterns (%)					
Breakfast	1904 (97.1)	1334 (97.4)	331 (97.4)	239 (95.6)	0.293
Mid-morning meal	1149 (58.6)	769 (56.1)	211 (62.1)	169 (67.6)	** 0.001 **
Lunch	1952 (99.6)	1366 (99.7)	338 (99.4)	248 (99.2)	0.434
Snack	891 (45.5)	602 (43.9)	164 (48.2)	125 (50.0)	0.110
Dinner	1946 (99.3)	1362 (99.4)	338 (99.4)	246 (98.4)	0.205
Re-dinner	430 (21.9)	304 (22.2)	61 (17.9)	65 (26.0)	0.060
Olive oil as main cooking fat (%)	1900 (96.9)	1320 (96.4)	334 (98.2)	246 (98.4)	0.070
Tea consumption (%)	98 (5.0)	52 (3.8)	26 (7.6)	20 (8.0)	** 0.001 **
Reading food nutritional labels (%)	579 (29.6)	335 (24.5)	144 (42.4)	100 (40.0)	** <0.001 **
Commercial pastry consumption = No (%)	1166 (59.5)	863 (63.0)	179 (52.6)	124 (49.6)	** <0.001 **
Preferably consume chicken, turkey, or rabbit meat (%)	768 (39.2)	513 (37.4)	150 (44.1)	105 (42.0)	** 0.049 **
**Cardiometabolic risk factors and related treatments**					
Hypertension (%)	868 (44.3)	661 (48.2)	116 (34.1)	91 (36.4)	** <0.001 **
Antihypertensive drugs (%)	546 (27.9)	430 (31.4)	66 (19.4)	50 (20.0)	** <0.001 **
Diabetes (%)	235 (12.0)	203 (14.8)	16 (4.7)	16 (6.4)	** <0.001 **
Hypoglycaemic drugs (%)	116 (5.9)	104 (7.6)	7 (2.1)	5 (2.0)	** <0.001 **
Dyslipidaemia (%)	1070 (54.6)	765 (55.8)	173 (50.9)	132 (52.8)	0.215
Lipid–lowering drugs (%)	458 (23.4)	358 (26.1)	56 (16.5)	44 (17.6)	** <0.001 **
**Other medical history**					
History of anaemia/bleeding (%)	411 (21.0)	269 (19.6)	90 (26.5)	52 (20.8)	** 0.021 **
Rheumatologic/immunologic disease (%)	160 (8.2)	108 (7.9)	29 (8.5)	23 (9.2)	0.755
Chronic kidney disease = Yes (%)	29 (1.5)	28 (2.0)	1 (0.3)	0 (0.0)	** 0.007 **
Cancer (%)	148 (7.6)	108 (7.9)	22 (6.5)	18 (7.2)	0.661
COPD (%)	57 (2.9)	48 (3.5)	5 (1.5)	4 (1.6)	0.057
Asthma (%)	145 (7.4)	100 (7.3)	22 (6.5)	23 (9.2)	0.442
Cognitive impairment/dementia (%)	15 (0.8)	13 (0.9)	2 (0.6)	0 (0.0)	0.262
Depression (%)	425 (21.7)	303 (22.1)	72 (21.2)	50 (20.0)	0.733
Anxiety (%)	804 (41.0)	545 (39.8)	151 (44.4)	108 (43.2)	0.226
**Cardiovascular disease history**					
History of any heart problem (%)	290 (14.8)	218 (15.9)	41 (12.1)	31 (12.4)	0.105
Stroke/TIA (%)	58 (3.0)	50 (3.6)	5 (1.5)	3 (1.2)	** 0.022 **
Heart failure (%)	71 (3.6)	55 (4.0)	14 (4.1)	2 (0.8)	** 0.038 **
Ischemic Heart Disease (%)	73 (3.7)	58 (4.2)	12 (3.5)	3 (1.2)	0.067
Arrhythmias (%)	142 (7.2)	112 (8.2)	17 (5.0)	13 (5.2)	0.053
Valvulopathy (%)	256 (13.1)	203 (14.8)	34 (10.1)	19 (7.6)	** 0.002 **
Coronary revascularization (%)	40 (2.1)	34 (2.5)	5 (1.5)	1 (0.4)	0.073
Aortic stenosis (%)					0.103
No	1876 (96.6)	1302 (95.8)	329 (97.9)	245 (98.8)	
Mild	52 (2.7)	45 (3.3)	4 (1.2)	3 (1.2)	
Moderate	9 (0.5)	8 (0.6)	1 (0.3)	0 (0.0)	
Severe	6 (0.3)	4 (0.3)	2 (0.6)	0 (0.0)	
**Overall medication use**					
Any medication (%)	877 (44.7)	643 (46.9)	133 (39.1)	101 (40.4)	0.012

BMI: body mass index; COPD: chronic obstructive pulmonary disease; DBP: diastolic blood pressure; IQR: interquartile range; SBP: systolic blood pressure; TIA: transient ischemic attack. Values are presented as median [IQR] or n (%). Non-parametric Kruskal–Wallis tests or chi-square tests were used, as appropriate. The bold is used to indicate *p*-values < 0.05.

**Table 2:** Biochemical parameters of participants according to frequency of chocolate consumption: no consumption, occasional consumption (1–3 times/week), and regular consumption (≥4 times/week).

	Overall	No Consumption	Occasional	Regular	*p*-Value
N	1960	1370	340	250	
Glucose (mg/dL) [median (IQR)]	90.00 [81.00, 100.00]	90.00 [81.00, 102.00]	89.00 [82.00, 96.00]	89.00 [81.00, 97.00]	** 0.021 **
HbA1c (%)	5.40 [5.20, 5.60]	5.40 [5.20, 5.70]	5.30 [5.10, 5.60]	5.30 [5.10, 5.53]	** <0.001 **
Total cholesterol (mg/dL) [median (IQR)]	182.00 [160.00, 201.00]	180.00 [159.00, 199.00]	180.00 [158.00, 206.00]	189.00 [167.00, 205.25]	** 0.043 **
HDL cholesterol (mg/dL) [median (IQR)]	52.10 [44.10, 61.65]	51.10 [43.12, 60.00]	53.30 [45.20, 62.23]	55.90 [47.30, 66.90]	** <0.001 **
LDL cholesterol (mg/dL) [median (IQR)]	104.88 [86.76, 122.42]	104.42 [86.87, 121.61]	104.73 [85.72, 123.70]	108.52 [89.64, 123.00]	0.514
Triglycerides (mg/dL) [median (IQR)]	97.40 [70.70, 138.70]	101.10 [73.40, 142.30]	92.25 [67.20, 135.72]	91.70 [66.75, 117.08]	** 0.011 **
hs-TnT (ng/L) [median (IQR)]	4.84 [3.00, 7.96]	5.34 [3.20, 8.79]	3.83 [3.00, 6.02]	3.87 [3.00, 6.81]	** <0.001 **
NT-proBNP (pg/mL) [median (IQR)]	50.52 [26.33, 95.35]	51.95 [26.44, 107.05]	47.17 [27.44, 82.09]	48.03 [24.25, 77.02]	0.056
hs-CRP (mg/dL) [median (IQR)]	0.12 [0.07, 0.28]	0.13 [0.07, 0.31]	0.11 [0.07, 0.27]	0.09 [0.05, 0.18]	** <0.001 **
GDF15 (pg/mL) [median (IQR)]	681.30 [497.70, 1021.00]	732.80 [519.50, 1100.50]	597.50 [469.50, 812.70]	610.60 [477.60, 878.40]	** <0.001 **
Creatinine (mg/dL) [median (IQR)]	0.83 [0.71, 0.95]	0.83 [0.72, 0.96]	0.82 [0.68, 0.93]	0.79 [0.72, 0.90]	** 0.044 **
Ferritin (ng/mL) [median (IQR)]	134.50 [60.00, 243.00]	148.00 [67.00, 263.00]	122.00 [54.75, 226.50]	113.00 [51.00, 202.00]	** 0.003 **
Transferrin (mg/mL) [median (IQR)]	245.00 [225.00, 269.00]	246.00 [223.75, 269.00]	244.00 [226.75, 266.00]	247.00 [228.00, 277.00]	0.392

GDF15: Growth Differentiation Factor-15; HbA1c: glycated haemoglobin; hs-CRP: high-sensitivity C-reactive protein; hs-TnT: high-sensitivity troponin T; HDL: high-density lipoprotein; IQR: interquartile range; LDL: low-density lipoprotein; NT-proBNP: *N*-terminal pro-brain natriuretic peptide; Values are presented as median [IQR] or n (%). Non-parametric Kruskal–Wallis tests or chi-square tests were used, as appropriate. The bold is used to indicate *p*-values < 0.05.

**Table 3:** *p*-values for interactions between chocolate type (dark and milk) and metabolic conditions in multivariable linear regression models for cardiometabolic risk markers.

Outcome	Dark Chocolate × Diabetes	Dark Chocolate × Hypertension	Dark Chocolate × Dyslipidaemia	Milk Chocolate × Diabetes	Milk Chocolate × Hypertension	Milk Chocolate × Dyslipidaemia
Glucose	<0.001 *** (<0.001 ^†††^)	0.874	0.207	0.014 * (0.652)	0.500	0.181
HbA1c	<0.001 *** (0.001 ^†††^)	0.688	0.464	<0.001 *** (0.009 ^††^)	0.271	0.542
HDL	0.163	0.609	0.215	0.481	0.815	0.030 *
LDL	0.722	0.092	0.996	0.400	0.448	0.158
TG	0.783	0.419	0.674	0.396	0.937	0.733
BMI	0.423	0.220	0.253	0.541	0.117	0.423
Waist	0.301	0.220	0.552	0.927	0.028 *	0.756
SBP	0.269	0.144	0.658	0.034 *	0.610	0.442

Values are shown as unadjusted *p*-values, with Bonferroni-adjusted *p*-values (pBonferroni) in parentheses; Bonferroni-adjusted values reaching 1 are omitted. Reference category: no consumption. Models were adjusted for age, sex, body mass index (BMI; except when BMI was the outcome), smoking status, daily alcohol consumption, physical activity level, olive oil intake, and commercial pastry consumption. Unadjusted *p*-values: * *p* < 0.05; *** *p* < 0.001. Bonferroni-adjusted *p*-values: ^††^ pBonferroni < 0.01; ^†††^ pBonferroni < 0.001. Bonferroni correction was applied across 48 interaction tests (8 outcomes × 2 chocolate types × 3 metabolic conditions), corresponding to an unadjusted significance threshold of α = 0.05/48 = 0.0010417. HbA1c, glycated haemoglobin; HDL, high-density lipoprotein cholesterol; LDL, low-density lipoprotein cholesterol; TG, triglycerides; SBP, systolic blood pressure.

**Table 4:** *p*-values for interaction between chocolate consumption frequency (regular vs. occasional consumption) and metabolic conditions in multivariable linear regression models for cardiometabolic risk markers.

Outcome	Dark Chocolate × Diabetes	Dark Chocolate × Hypertension	Dark Chocolate × Dyslipidaemia	Milk Chocolate × Diabetes	Milk Chocolate × Hypertension	Milk Chocolate × Dyslipidaemia
Glucose	0.028 *	0.860	0.853	0.244	0.808	0.251
HbA1c	0.271	0.271	0.696	0.442	0.668	0.304
HDL	0.134	0.101	0.780	0.653	0.950	0.565
LDL	0.008 ** (0.364)	0.828	0.178	0.214	0.118	0.884
TG	0.034 *	0.106	0.978	0.520	0.956	0.163
BMI	0.320	0.578	0.781	0.268	0.495	0.228
Waist	0.242	0.277	0.668	0.986	0.353	0.808
SBP	0.618	0.755	0.647	0.988	0.656	0.318

Values are shown as unadjusted *p*-values, with Bonferroni-adjusted *p*-values (pBonferroni) in parentheses; Bonferroni-adjusted values reaching 1 are omitted. Bonferroni correction was applied across 48 interaction tests (8 outcomes × 2 chocolate consumption types × 3 metabolic conditions), corresponding to an unadjusted significance threshold of α = 0.05/48 = 0.0010417. Reference category: occasional consumption of the corresponding chocolate type. Models were adjusted for age, sex, body mass index (BMI; except when BMI was the outcome), smoking status, daily alcohol consumption, physical activity level, olive oil intake, and commercial pastry consumption. Unadjusted *p*-values: * *p* < 0.05; ** *p* < 0.01. No interaction term reached statistical significance after Bonferroni correction. BMI, body mass index; HbA1c, glycated haemoglobin; HDL, high-density lipoprotein cholesterol; LDL, low-density lipoprotein cholesterol; TG, triglycerides; SBP, systolic blood pressure.

## Data Availability

The data presented in this study are available on request from the corresponding authors.

## Supplementary Materials

The following supporting information can be downloaded at: https://www.mdpi.com/article/10.3390/nu18183023/s1, Table S1. Complete results of multivariable linear regression models evaluating the association between chocolate consumption and cardiometabolic risk markers, including interaction terms with diabetes, hypertension, and dyslipidaemia; Table S2A. Complete results of multivariable linear regression models among dark chocolate consumers evaluating the associations between dark chocolate consumption frequency (regular vs. occasional) and cardiometabolic risk markers; Table S2B. Complete results of multivariable linear regression models evaluating the associations between milk chocolate consumption frequency (regular vs. occasional) and cardiometabolic risk markers.

## Abbreviations

The following abbreviations are used in this manuscript:

BMI	Body mass index
CAVI	Cardio-Ankle Vascular Index
CI	Confidence interval
DBP	Diastolic blood pressure
ECLIA	Electrochemiluminescence immunoassay
GDF15	Growth differentiation factor 15
HbA1c	Glycated haemoglobin
HDL	High density lipoprotein
HPLC	High-performance liquid chromatography
hs-CRP	High-sensitivity C-reactive protein
hs-TnT	High-sensitivity troponin T
IL-6	Interleukin-6
ISE	Ion-selective electrodes
LDL	Low density lipoprotein
NF-κB	Nuclear factor kappa B
NT-proBNP	*N*-terminal pro-B-type natriuretic peptide
SBP	Systolic blood pressure
TG	Triglycerides
TIA	Transient ischemic attack
TNF-α	Tumour necrosis factor alpha
