# Supplementary material for: Associations Between Chocolate Consumption and Cardiometabolic Risk Markers Across Metabolic Profiles: Insights from a Population-Based Study
Source: Nutrients. 2026 Sep 16;18(18):3023. doi: 10.3390/nu18183023 (PMC13610764; doi:10.3390/nu18183023)
Supplement: Supplementary file 1 [file nutrients-18-03023-s001.zip › Table S1.pdf]

Table S1. Complete results of multivariable linear regression models evaluating the association between chocolate consumption and cardiometabolic risk markers, including interaction terms with diabetes, hypertension, and dyslipidaemia.

	Glucose			HbA1c			HDL.			LDL			TG			BMI			Waist			SBP		
Term	β (95%CI)	p	p Bonf.	β (95%CI)	p	p Bonf.	β (95%CI)	p	p Bonf.	β (95%CI)	p	p Bonf.	β (95%CI)	p	p Bonf.	β (95%CI)	p	p Bonf.	β (95%CI)	p	p Bonf.	β (95%CI)	p	p Bonf.
Intercept	-0.25 (-0.45,-0.04)	0.020*	—	-0.26 (-0.45,-0.07)	0.008**	—	-0.43 (-0.65,-0.20)	<0.001***	—	-0.15 (-0.40,0.10)	0.227	—	-0.29 (-0.52,-0.07)	0.010*	—	0.29 (0.12,0.45)	<0.001***	—	0.29 (0.19,0.39)	<0.001***	—	-0.54 (-0.68,-0.40)	<0.001***	—
Age (per SD)	0.07 (0.00,0.13)	0.047*	—	0.24 (0.18,0.30)	<0.001***	—	0.05 (-0.02,0.12)	0.177	—	0.02 (-0.06,0.09)	0.694	—	-0.08 (-0.15,-0.01)	0.018*	—	0.03 (-0.02,0.08)	0.269	—	0.03 (-0.00,0.06)	0.088	—	0.20 (0.16,0.24)	<0.001***	—
Sex (Women vs. Men)	-0.20 (-0.31,-0.09)	<0.001***	—	-0.01 (-0.10,0.09)	0.910	—	0.83 (0.72,0.95)	<0.001***	—	0.06 (-0.07,0.19)	0.348	—	-0.31 (-0.43,-0.20)	<0.001***	—	-0.28 (-0.37,-0.19)	<0.001***	—	-0.38 (-0.43,-0.32)	<0.001***	—	-0.02 (-0.09,0.05)	0.587	—
Current smoker (Yes vs. No)	-0.13 (-0.26,0.00)	0.056	—	0.21 (0.09,0.33)	<0.001***	—	-0.12 (-0.26,0.02)	0.099	—	0.09 (-0.06,0.25)	0.243	—	0.18 (0.04,0.32)	0.012*	—	-0.11 (-0.21,0.00)	0.052	—	-0.03 (-0.10,0.03)	0.315	—	0.04 (-0.05,0.13)	0.400	—
Daily alcohol consumption (Yes vs. No)	-0.02 (-0.14,0.10)	0.741	—	-0.10 (-0.21,0.01)	0.085	—	0.28 (0.15,0.42)	<0.001***	—	-0.02 (-0.17,0.13)	0.793	—	-0.06 (-0.20,0.07)	0.355	—	-0.05 (-0.16,0.05)	0.300	—	0.01 (-0.06,0.07)	0.860	—	0.04 (-0.04,0.13)	0.315	—
Physical activity: Moderate vs. Low/inactive	0.06 (-0.10,0.22)	0.437	—	0.03 (-0.11,0.17)	0.681	—	0.02 (-0.15,0.19)	0.853	—	-0.14 (-0.34,0.05)	0.145	—	-0.10 (-0.27,0.07)	0.235	—	-0.30 (-0.43,-0.17)	<0.001***	—	-0.11 (-0.19,-0.03)	0.005**	—	0.19 (0.08,0.29)	<0.001***	—
Physical activity: High vs. Low/inactive	0.04 (-0.20,0.27)	0.758	—	0.03 (-0.18,0.25)	0.754	—	0.14 (-0.12,0.39)	0.295	—	-0.20 (-0.48,0.08)	0.165	—	-0.21 (-0.47,0.04)	0.102	—	-0.56 (-0.75,-0.37)	<0.001***	—	-0.17 (-0.29,-0.05)	0.004**	—	0.23 (0.08,0.39)	0.003**	—
Commercial pastry <2/week (vs. No)	-0.06 (-0.16,0.04)	0.270	—	-0.06 (-0.15,0.03)	0.202	—	0.06 (-0.05,0.17)	0.308	—	-0.02 (-0.14,0.10)	0.785	—	-0.08 (-0.19,0.03)	0.173	—	0.01 (-0.08,0.10)	0.818	—	-0.03 (-0.08,0.02)	0.252	—	-0.02 (-0.09,0.05)	0.498	—
Olive oil ≥4 tbsp/day (vs. <4 tbsp)	-0.01 (-0.11,0.09)	0.851	—	-0.06 (-0.16,0.03)	0.171	—	0.03 (-0.08,0.14)	0.630	—	-0.02 (-0.14,0.09)	0.697	—	0.04 (-0.06,0.15)	0.420	—	-0.04 (-0.12,0.05)	0.417	—	-0.02 (-0.07,0.03)	0.475	—	-0.07 (-0.14,-0.00)	0.050*	—
BMI (per SD)	0.02 (-0.03,0.08)	0.395	—	0.13 (0.08,0.18)	<0.001***	—	-0.19 (-0.25,-0.13)	<0.001***	—	0.08 (0.02,0.15)	0.008**	—	0.16 (0.10,0.22)	<0.001***	—				0.74 (0.72,0.77)	<0.001***	—	0.13 (0.10,0.17)	<0.001***	—
Dark chocolate (Yes vs. No)	0.12 (-0.10,0.34)	0.282	—	0.04 (-0.16,0.25)	0.661	—	0.04 (-0.20,0.27)	0.771	—	-0.05 (-0.30,0.20)	0.716	—	0.07 (-0.16,0.31)	0.537	—	-0.19 (-0.36,-0.01)	0.042*	—	0.01 (-0.10,0.12)	0.843	—	-0.01 (-0.15,0.14)	0.901	—
Milk chocolate (Yes vs. No)	0.19 (-0.04,0.41)	0.106	—	0.01 (-0.19,0.22)	0.897	—	-0.17 (-0.42,0.07)	0.164	—	-0.29 (-0.55,-0.04)	0.026*	—	-0.04 (-0.29,0.20)	0.741	—	-0.33 (-0.52,-0.14)	<0.001***	—	-0.03 (-0.15,0.08)	0.591	—	-0.06 (-0.21,0.10)	0.477	—
Diabetes (Yes vs. No)	1.77 (1.58,1.95)	<0.001***	—	1.77 (1.60,1.93)	<0.001***	—	-0.16 (-0.36,0.04)	0.109	—	-0.74 (-0.96,-0.52)	<0.001***	—	0.21 (0.01,0.40)	0.038*	—	0.24 (0.09,0.38)	0.001**	—	0.09 (0.00,0.18)	0.039*	—	-0.13 (-0.25,-0.01)	0.029*	—
Hypertension (Yes vs. No)	0.18 (0.04,0.32)	0.010*	—	0.06 (-0.06,0.18)	0.341	—	-0.14 (-0.28,0.01)	0.074	—	-0.17 (-0.34,-0.01)	0.037*	—	0.19 (0.04,0.33)	0.014*	—	0.40 (0.28,0.51)	<0.001***	—	0.10 (0.04,0.17)	0.003**	—	0.96 (0.87,1.05)	<0.001***	—
Dyslipidaemia (Yes vs. No)	0.19 (0.06,0.32)	0.004**	—	0.16 (0.05,0.28)	0.006**	—	-0.16 (-0.29,-0.02)	0.029*	—	0.73 (0.58,0.88)	<0.001***	—	0.79 (0.65,0.93)	<0.001***	—	0.09 (-0.01,0.19)	0.083	—	-0.03 (-0.09,0.03)	0.326	—	-0.00 (-0.08,0.08)	0.990	—
**Dark chocolate × Diabetes**	-1.64 (-2.16,-1.13)	**<0.001*****	**<0.001†††**	-1.00 (-1.46,-0.53)	**<0.001*****	**0.001††**	-0.40 (-0.95,0.16)	0.163	1.000	0.12 (-0.52,0.76)	0.722	1.000	0.08 (-0.48,0.63)	0.783	1.000	0.18 (-0.26,0.62)	0.423	1.000	0.14 (-0.13,0.41)	0.301	1.000	0.20 (-0.16,0.56)	0.269	1.000
**Dark chocolate × Hypertension**	0.02 (-0.25,0.29)	0.874	1.000	0.05 (-0.19,0.29)	0.688	1.000	-0.08 (-0.37,0.21)	0.609	1.000	0.27 (-0.04,0.59)	0.092	1.000	-0.12 (-0.41,0.17)	0.419	1.000	0.15 (-0.09,0.38)	0.220	1.000	-0.09 (-0.23,0.05)	0.220	1.000	0.14 (-0.05,0.34)	0.144	1.000
**Dark chocolate × Dyslipidaemia**	-0.18 (-0.45,0.10)	0.207	1.000	-0.09 (-0.34,0.16)	0.464	1.000	0.19 (-0.11,0.48)	0.215	1.000	0.00 (-0.31,0.32)	0.996	1.000	-0.06 (-0.36,0.23)	0.674	1.000	-0.14 (-0.37,0.10)	0.253	1.000	0.04 (-0.10,0.18)	0.552	1.000	-0.04 (-0.23,0.15)	0.658	1.000
**Milk chocolate × Diabetes**	-0.89 (-1.59,-0.18)	0.014*	0.652	-1.23 (-1.87,-0.59)	**<0.001*****	**0.009††**	-0.27 (-1.04,0.49)	0.481	1.000	0.37 (-0.50,1.24)	0.400	1.000	-0.33 (-1.09,0.43)	0.396	1.000	-0.20 (-0.82,0.43)	0.541	1.000	-0.02 (-0.39,0.36)	0.927	1.000	0.55 (0.04,1.06)	0.034*	1.000
**Milk chocolate × Hypertension**	-0.11 (-0.42,0.21)	0.500	1.000	0.16 (-0.12,0.44)	0.271	1.000	0.04 (-0.30,0.38)	0.815	1.000	0.15 (-0.23,0.52)	0.448	1.000	0.01 (-0.32,0.35)	0.937	1.000	0.22 (-0.06,0.50)	0.117	1.000	0.19 (0.02,0.35)	0.028*	1.000	-0.06 (-0.28,0.17)	0.610	1.000
**Milk chocolate × Dyslipidaemia**	-0.20 (-0.48,0.09)	0.181	1.000	-0.08 (-0.34,0.18)	0.542	1.000	0.35 (0.03,0.66)	0.030*	1.000	0.25 (-0.10,0.59)	0.158	1.000	0.05 (-0.26,0.37)	0.733	1.000	0.10 (-0.15,0.36)	0.423	1.000	0.02 (-0.13,0.18)	0.756	1.000	-0.08 (-0.29,0.12)	0.442	1.000
**Model fit**																								
n	1,042			1,040			1,043			950			1,041			1,961			1,950			1,952		
R^2	0.371			0.482			0.261			0.200			0.258			0.150			0.692			0.441		
Adjusted R^2	0.359			0.472			0.246			0.183			0.243			0.142			0.689			0.435		

β: standardised regression coefficient (outcomes scaled to z-scores). 95% confidence interval (CI) in parentheses.
p: uncorrected p-value. p Bonf.: Bonferroni-corrected p-value, reported for interaction terms; — = not applicable.
R^2: coefficient of determination. Adjusted R^2: R^2 penalised for number of predictors. n: observations with complete data.
Uncorrected p-values: * $p<0.05$; ** $p<0.01$; *** $p<0.001$. Bonferroni-corrected p-values: † $p<0.05$; †† $p<0.01$; ††† $p<0.001$.
Models were adjusted for age, sex, body mass index (BMI; except when BMI was the outcome), smoking status, daily alcohol consumption, physical activity level, olive oil intake, and commercial pastry consumption.
Interaction terms represent effect modification of the association between each chocolate type and each cardiometabolic outcome according to the presence of diabetes, hypertension, or dyslipidaemia, while simultaneously adjusting for the other two metabolic conditions.
HbA1c, glycated haemoglobin; HDL, high-density lipoprotein cholesterol; LDL, low-density lipoprotein cholesterol; TG, triglycerides; SBP, systolic blood pressure
