# Supplementary material for: Associations Between Chocolate Consumption and Cardiometabolic Risk Markers Across Metabolic Profiles: Insights from a Population-Based Study
Source: Nutrients. 2026 Sep 16;18(18):3023. doi: 10.3390/nu18183023 (PMC13610764; doi:10.3390/nu18183023)
Supplement: Supplementary file 1 [file nutrients-18-03023-s001.zip › Table S2.pdf]

Table S2A. Complete results of multivariable linear regression models among dark chocolate consumers evaluating the associations between dark chocolate consumption frequency (regular vs. occasional) and cardiometabolic risk markers.

Term	Glucose			HbA1c			HDL			LDL			TG			BMI			Waist			SBP		
Term	β (95%CI)	p	p Bonf.	β (95%CI)	p	p Bonf.	β (95%CI)	p	p Bonf.	β (95%CI)	p	p Bonf.	β (95%CI)	p	p Bonf.	β (95%CI)	p	p Bonf.	β (95%CI)	p	p Bonf.	β (95%CI)	p	p Bonf.
Intercept	-0.24 (-0.59,0.10)	0.171	—	-0.44 (-0.79,-0.08)	0.017*	—	-0.63 (-1.26,0.00)	0.050	—	-0.68 (-1.34,-0.02)	0.043*	—	-0.38 (-0.99,0.24)	0.232	—	-0.01 (-0.42,0.40)	0.968	—	0.14 (-0.10,0.38)	0.262	—	-0.49 (-0.84,-0.14)	0.006**	—
Age (per SD)	0.07 (-0.04,0.19)	0.204	—	0.32 (0.20,0.44)	<0.001***	—	-0.09 (-0.30,0.12)	0.386	—	0.02 (-0.19,0.23)	0.826	—	-0.01 (-0.21,0.19)	0.942	—	-0.08 (-0.21,0.05)	0.210	—	0.09 (0.01,0.17)	0.020*	—	0.21 (0.10,0.31)	<0.001***	—
Sex (Women vs. Men)	-0.03 (-0.20,0.14)	0.746	—	0.06 (-0.11,0.24)	0.495	—	0.79 (0.48,1.10)	<0.001***	—	0.13 (-0.20,0.45)	0.445	—	-0.12 (-0.43,0.18)	0.427	—	-0.23 (-0.44,-0.03)	0.025*	—	-0.43 (-0.56,-0.31)	<0.001***	—	-0.08 (-0.26,0.09)	0.341	—
Current smoker (Yes vs. No)	-0.33 (-0.53,-0.14)	0.001**	—	0.08 (-0.12,0.29)	0.417	—	-0.01 (-0.37,0.35)	0.944	—	-0.06 (-0.43,0.31)	0.752	—	0.03 (-0.32,0.39)	0.849	—	-0.08 (-0.33,0.17)	0.524	—	0.07 (-0.08,0.22)	0.349	—	0.03 (-0.18,0.24)	0.781	—
Daily alcohol consumption (Yes vs. No)	0.10 (-0.10,0.30)	0.315	—	0.05 (-0.15,0.26)	0.606	—	0.15 (-0.21,0.51)	0.418	—	0.20 (-0.18,0.58)	0.296	—	-0.15 (-0.50,0.20)	0.387	—	0.13 (-0.10,0.36)	0.259	—	-0.06 (-0.19,0.08)	0.416	—	-0.01 (-0.20,0.19)	0.952	—
Physical activity: Moderate vs. Low/inactive	0.00 (-0.28,0.28)	1.000	—	0.14 (-0.15,0.43)	0.340	—	0.04 (-0.47,0.55)	0.870	—	0.27 (-0.28,0.81)	0.335	—	-0.00 (-0.51,0.50)	0.987	—	-0.20 (-0.54,0.14)	0.240	—	0.12 (-0.08,0.32)	0.254	—	0.18 (-0.11,0.47)	0.230	—
Physical activity: High vs. Low/inactive	0.08 (-0.31,0.46)	0.697	—	0.33 (-0.07,0.72)	0.106	—	-0.24 (-0.94,0.45)	0.492	—	0.39 (-0.33,1.11)	0.289	—	0.06 (-0.62,0.74)	0.860	—	-0.63 (-1.07,-0.19)	0.005**	—	0.25 (-0.01,0.52)	0.059	—	0.40 (0.02,0.79)	0.037*	—
Commercial pastry <2/week (vs. No)	0.04 (-0.11,0.19)	0.607	—	0.07 (-0.09,0.22)	0.402	—	-0.06 (-0.34,0.21)	0.647	—	-0.18 (-0.46,0.09)	0.195	—	0.11 (-0.16,0.38)	0.424	—	-0.04 (-0.22,0.14)	0.666	—	-0.02 (-0.13,0.09)	0.682	—	0.06 (-0.09,0.21)	0.441	—
Olive oil ≥4 tbsp/day (vs. <4 tbsp)	0.14 (-0.01,0.28)	0.067	—	-0.00 (-0.15,0.15)	0.972	—	0.26 (-0.01,0.52)	0.055	—	-0.16 (-0.44,0.11)	0.236	—	0.09 (-0.17,0.34)	0.518	—	-0.07 (-0.25,0.12)	0.488	—	-0.05 (-0.16,0.06)	0.398	—	-0.05 (-0.21,0.10)	0.499	—
BMI (per SD)	0.03 (-0.07,0.12)	0.589	—	0.14 (0.04,0.25)	0.006**	—	-0.35 (-0.53,-0.17)	<0.001***	—	-0.05 (-0.24,0.13)	0.573	—	0.31 (0.14,0.49)	<0.001***	—				0.75 (0.69,0.82)	<0.001***	—	0.17 (0.07,0.26)	<0.001***	—
Regular consumption (vs. Occasional)	-0.09 (-0.34,0.16)	0.460	—	0.03 (-0.23,0.29)	0.820	—	0.15 (-0.30,0.61)	0.505	—	0.21 (-0.24,0.67)	0.350	—	-0.07 (-0.51,0.38)	0.764	—	-0.11 (-0.39,0.18)	0.462	—	-0.02 (-0.19,0.15)	0.795	—	-0.14 (-0.39,0.10)	0.248	—
Diabetes (Yes vs. No)	-0.25 (-0.69,0.19)	0.265	—	0.57 (0.11,1.02)	0.016*	—	-0.92 (-1.72,-0.11)	0.026*	—	-1.82 (-2.82,-0.82)	<0.001***	—	0.91 (0.12,1.70)	0.024*	—	0.55 (0.03,1.07)	0.040*	—	0.11 (-0.20,0.42)	0.472	—	-0.02 (-0.47,0.43)	0.933	—
Hypertension (Yes vs. No)	0.21 (-0.02,0.44)	0.073	—	0.11 (-0.12,0.35)	0.344	—	0.20 (-0.22,0.62)	0.359	—	0.14 (-0.29,0.57)	0.526	—	-0.23 (-0.64,0.18)	0.278	—	0.66 (0.39,0.94)	<0.001***	—	-0.09 (-0.26,0.08)	0.313	—	1.05 (0.80,1.29)	<0.001***	—
Dyslipidaemia (Yes vs. No)	-0.01 (-0.23,0.21)	0.915	—	0.02 (-0.21,0.24)	0.892	—	0.08 (-0.31,0.48)	0.675	—	0.96 (0.56,1.37)	<0.001***	—	0.63 (0.24,1.02)	0.002**	—	0.03 (-0.24,0.29)	0.844	—	0.04 (-0.12,0.19)	0.638	—	-0.04 (-0.27,0.18)	0.701	—
**Regular × Diabetes**	**0.69 (0.07,1.31)**	**0.028***	***1.000***	**0.36 (-0.28,1.00)**	**0.271**	***1.000***	**0.86 (-0.27,1.98)**	**0.134**	***1.000***	**1.73 (0.47,2.99)**	**0.008****	***0.364***	**-1.19 (-2.29,-0.09)**	**0.034***	***1.000***	**-0.38 (-1.12,0.37)**	**0.320**	***1.000***	**0.26 (-0.18,0.70)**	**0.242**	***1.000***	**0.16 (-0.47,0.79)**	**0.618**	***1.000***
**Regular × Hypertension**	**-0.03 (-0.34,0.28)**	**0.860**	***1.000***	**-0.18 (-0.50,0.14)**	**0.271**	***1.000***	**-0.47 (-1.04,0.09)**	**0.101**	***1.000***	**0.07 (-0.53,0.66)**	**0.828**	***1.000***	**0.46 (-0.10,1.01)**	**0.106**	***1.000***	**-0.11 (-0.50,0.28)**	**0.578**	***1.000***	**0.13 (-0.10,0.36)**	**0.277**	***1.000***	**0.05 (-0.28,0.38)**	**0.755**	***1.000***
**Regular × Dyslipidaemia**	**0.03 (-0.28,0.34)**	**0.853**	***1.000***	**0.07 (-0.26,0.39)**	**0.696**	***1.000***	**0.08 (-0.49,0.66)**	**0.780**	***1.000***	**-0.40 (-0.98,0.18)**	**0.178**	***1.000***	**-0.01 (-0.57,0.55)**	**0.978**	***1.000***	**-0.05 (-0.43,0.32)**	**0.781**	***1.000***	**-0.05 (-0.27,0.17)**	**0.668**	***1.000***	**0.07 (-0.25,0.40)**	**0.647**	***1.000***
**Model fit**																								
n	*202*			*201*			*202*			*189*			*202*			*335*			*332*			*334*		
R²	*0.210*			*0.368*			*0.306*			*0.228*			*0.243*			*0.197*			*0.717*			*0.513*		
Adjusted R²	*0.142*			*0.313*			*0.246*			*0.156*			*0.177*			*0.160*			*0.703*			*0.489*		

Table S2B. Complete results of multivariable linear regression models evaluating the associations between milk chocolate consumption frequency (regular vs. occasional) and cardiometabolic risk markers.

Term	Glucose			HbA1c			HDL			LDL			TG			BMI			Waist			SBP		
Term	β (95%CI)	p	p Bonf.	β (95%CI)	p	p Bonf.	β (95%CI)	p	p Bonf.	β (95%CI)	p	p Bonf.	β (95%CI)	p	p Bonf.	β (95%CI)	p	p Bonf.	β (95%CI)	p	p Bonf.	β (95%CI)	p	p Bonf.
Intercept	-0.20 (-0.61,0.21)	0.331	—	-0.44 (-0.84,-0.03)	0.036*	—	-0.61 (-1.29,0.08)	0.085	—	-0.76 (-1.38,-0.15)	0.016*	—	-0.59 (-1.29,0.12)	0.102	—	0.73 (0.27,1.20)	0.002**	—	0.45 (0.18,0.72)	0.001**	—	-0.73 (-1.13,-0.33)	<0.001***	—
Age (per SD)	0.12 (0.00,0.24)	0.046*	—	0.26 (0.14,0.37)	<0.001***	—	0.17 (-0.02,0.37)	0.084	—	-0.03 (-0.21,0.14)	0.718	—	-0.22 (-0.42,-0.01)	0.036*	—	0.22 (0.08,0.35)	0.002**	—	0.04 (-0.04,0.12)	0.285	—	0.20 (0.08,0.32)	0.001**	—
Sex (Women vs. Men)	-0.05 (-0.25,0.14)	0.593	—	-0.01 (-0.20,0.19)	0.926	—	0.74 (0.40,1.07)	<0.001***	—	0.12 (-0.18,0.41)	0.432	—	-0.23 (-0.57,0.11)	0.177	—	-0.55 (-0.79,-0.32)	<0.001***	—	-0.37 (-0.51,-0.23)	<0.001***	—	-0.10 (-0.31,0.11)	0.337	—
Current smoker (Yes vs. No)	-0.09 (-0.32,0.14)	0.445	—	0.07 (-0.16,0.30)	0.555	—	0.02 (-0.37,0.42)	0.902	—	0.16 (-0.20,0.51)	0.378	—	0.44 (0.04,0.85)	0.030*	—	-0.29 (-0.56,-0.03)	0.032*	—	-0.17 (-0.32,-0.01)	0.032*	—	0.23 (-0.00,0.46)	0.053	—
Daily alcohol consumption (Yes vs. No)	-0.23 (-0.50,0.04)	0.099	—	-0.25 (-0.53,0.02)	0.074	—	0.24 (-0.21,0.70)	0.293	—	-0.22 (-0.62,0.18)	0.278	—	0.11 (-0.35,0.58)	0.634	—	-0.52 (-0.84,-0.20)	0.002**	—	0.06 (-0.12,0.25)	0.494	—	-0.24 (-0.52,0.04)	0.091	—
Physical activity: Moderate vs. Low/inactive	0.16 (-0.14,0.46)	0.292	—	0.26 (-0.03,0.56)	0.081	—	-0.10 (-0.60,0.41)	0.707	—	0.06 (-0.39,0.51)	0.788	—	0.05 (-0.46,0.56)	0.843	—	-0.59 (-0.92,-0.26)	<0.001***	—	-0.21 (-0.40,-0.02)	0.030*	—	0.23 (-0.05,0.52)	0.111	—
Physical activity: High vs. Low/inactive	0.19 (-0.22,0.59)	0.362	—	0.36 (-0.05,0.76)	0.081	—	0.26 (-0.42,0.95)	0.450	—	-0.03 (-0.65,0.60)	0.935	—	0.15 (-0.56,0.86)	0.680	—	-0.91 (-1.40,-0.41)	<0.001***	—	-0.46 (-0.75,-0.17)	0.002**	—	0.48 (0.05,0.91)	0.030*	—
Commercial pastry <2/week (vs. No)	0.05 (-0.13,0.22)	0.594	—	-0.03 (-0.20,0.15)	0.767	—	0.05 (-0.25,0.34)	0.744	—	0.11 (-0.15,0.37)	0.397	—	-0.22 (-0.52,0.08)	0.154	—	-0.01 (-0.22,0.21)	0.937	—	-0.05 (-0.18,0.07)	0.389	—	-0.04 (-0.22,0.14)	0.679	—
Olive oil ≥4 tbsp/day (vs. <4 tbsp)	-0.19 (-0.36,-0.02)	0.033*	—	-0.13 (-0.31,0.04)	0.132	—	0.11 (-0.18,0.41)	0.446	—	-0.14 (-0.39,0.12)	0.296	—	-0.12 (-0.42,0.18)	0.424	—	0.06 (-0.15,0.27)	0.587	—	0.01 (-0.11,0.13)	0.835	—	0.13 (-0.05,0.31)	0.156	—
BMI (per SD)	0.05 (-0.06,0.16)	0.352	—	0.19 (0.08,0.29)	<0.001***	—	-0.33 (-0.52,-0.15)	<0.001***	—	0.19 (0.03,0.35)	0.018*	—	0.28 (0.10,0.47)	0.003**	—				0.84 (0.77,0.92)	<0.001***	—	0.21 (0.10,0.32)	<0.001***	—
Regular consumption (vs. Occasional)	0.12 (-0.15,0.39)	0.375	—	0.25 (-0.02,0.51)	0.068	—	0.30 (-0.15,0.75)	0.188	—	0.25 (-0.14,0.63)	0.208	—	0.03 (-0.43,0.50)	0.885	—	-0.09 (-0.40,0.22)	0.576	—	0.05 (-0.12,0.23)	0.544	—	0.19 (-0.07,0.45)	0.158	—
Diabetes (Yes vs. No)	1.00 (0.42,1.57)	<0.001***	—	0.38 (-0.19,0.95)	0.191	—	-0.54 (-1.51,0.42)	0.266	—	-0.70 (-1.64,0.23)	0.137	—	0.04 (-0.94,1.02)	0.941	—	-0.24 (-1.02,0.53)	0.537	—	0.07 (-0.37,0.52)	0.743	—	0.45 (-0.21,1.11)	0.177	—
Hypertension (Yes vs. No)	0.19 (-0.08,0.45)	0.161	—	0.20 (-0.07,0.46)	0.142	—	-0.12 (-0.57,0.33)	0.590	—	0.22 (-0.19,0.62)	0.286	—	0.33 (-0.12,0.79)	0.151	—	0.30 (-0.03,0.62)	0.072	—	0.19 (0.00,0.38)	0.044*	—	0.92 (0.64,1.20)	<0.001***	—
Dyslipidaemia (Yes vs. No)	0.05 (-0.19,0.29)	0.695	—	0.11 (-0.14,0.35)	0.389	—	0.26 (-0.15,0.67)	0.212	—	1.03 (0.67,1.40)	<0.001***	—	1.05 (0.64,1.47)	<0.001***	—	0.17 (-0.11,0.44)	0.229	—	-0.05 (-0.21,0.10)	0.499	—	0.01 (-0.22,0.24)	0.944	—
**Regular × Diabetes**	**-0.59 (-1.59,0.41)**	**0.244**	***1.000***	**0.39 (-0.61,1.39)**	**0.442**	***1.000***	**0.39 (-1.30,2.07)**	**0.653**	***1.000***	**0.95 (-0.56,2.46)**	**0.214**	***1.000***	**-0.56 (-2.28,1.16)**	**0.520**	***1.000***	**0.64 (-0.50,1.78)**	**0.268**	***1.000***	**-0.01 (-0.66,0.64)**	**0.986**	***1.000***	**-0.01 (-0.98,0.96)**	**0.988**	***1.000***
**Regular × Hypertension**	**-0.05 (-0.47,0.36)**	**0.808**	***1.000***	**0.09 (-0.32,0.50)**	**0.668**	***1.000***	**0.02 (-0.68,0.72)**	**0.950**	***1.000***	**-0.50 (-1.13,0.13)**	**0.118**	***1.000***	**-0.02 (-0.73,0.69)**	**0.956**	***1.000***	**0.17 (-0.33,0.68)**	**0.495**	***1.000***	**-0.14 (-0.42,0.15)**	**0.353**	***1.000***	**-0.10 (-0.53,0.33)**	**0.656**	***1.000***
**Regular × Dyslipidaemia**	**-0.22 (-0.59,0.16)**	**0.251**	***1.000***	**-0.19 (-0.57,0.18)**	**0.304**	***1.000***	**-0.18 (-0.82,0.45)**	**0.565**	***1.000***	**-0.04 (-0.59,0.51)**	**0.884**	***1.000***	**-0.46 (-1.11,0.19)**	**0.163**	***1.000***	**-0.28 (-0.73,0.17)**	**0.228**	***1.000***	**0.03 (-0.23,0.29)**	**0.808**	***1.000***	**-0.19 (-0.58,0.19)**	**0.318**	***1.000***
**Model fit**																								
n	*160*			*160*			*160*			*144*			*159*			*257*			*257*			*257*		
R^2	*0.236*			*0.428*			*0.303*			*0.385*			*0.352*			*0.314*			*0.816*			*0.463*		
Adjusted R^2	*0.151*			*0.364*			*0.225*			*0.308*			*0.279*			*0.272*			*0.803*			*0.427*		

β: standardised regression coefficient (outcomes scaled to z-scores). 95% confidence interval (CI) in parentheses.
p: uncorrected p-value. p Bonf.: Bonferroni-corrected p-value, reported for interaction terms; — = not applicable.
Uncorrected p-values: * p<0.05; ** p<0.01; *** p<0.001. Bonferroni-corrected p-values: † p<0.05; †† p<0.01; ††† p<0.001.
Models were adjusted for age, sex, body mass index (BMI; except when BMI was the outcome), smoking status, daily alcohol consumption, physical activity level, olive oil intake, and commercial pastry consumption.
Reference category: occasional consumption (1–3 days/week).
Interaction terms represent effect modification of the association between chocolate consumption frequency and each cardiometabolic outcome according to the presence of diabetes, hypertension, or dyslipidaemia, while simultaneously adjusting for the other two metabolic conditions.
BMI, body mass index; HbA1c, glycated haemoglobin; HDL, high-density lipoprotein cholesterol; LDL, low-density lipoprotein cholesterol; TG, triglycerides; SBP, systolic blood pressure.
